# 1225. The Perfect Storm: A Hardy and Lethal Pathogen and a Unit Filled with Immunocompromised Patients with Large Open Wounds. Troubles with Candida Auris in a Burn Intensive Care Unit

**DOI:** 10.1093/ofid/ofac492.1057

**Published:** 2022-12-15

**Authors:** Riley Moore, Louise Lie, Herminia Pua, David H Slade, Sharon Rangel, Anthony Davila, Joshua S Carson, Jorge Paiva Parada

**Affiliations:** Loyola University Medical Center, Maywood, Illinois; Loyola University Medical Center, Maywood, Illinois; Loyola University Medical Center, Maywood, Illinois; Loyola University Medical Center, Maywood, Illinois; Loyola University Medical Center, Maywood, Illinois; Loyola University Medical Center, Maywood, Illinois; Loyola University Medical Center, Maywood, Illinois; Loyola University Chicago, La Grange, Illinois

## Abstract

**Background:**

*C auris* is an emerging often multidrug resistant pathogen capable of causing severe morbidity and mortality. *C auris* has been increasingly isolated from patients in skilled nursing facilities and hospitals, and has been associated with facility outbreaks. A resilient pathogen, *C. auris* survives harsh disinfectants, desiccation and readily colonizes the environment posing an especially great risk to immunocompromised patients with large open wounds and long lengths of stay.

**Methods:**

From 7/1/21 - 8/30/21 we noted a cluster of 4 patients with *C auris* in clinical cultures from the Burn ICU (BICU). A multidisciplinary team involving infection prevention, nursing/medical directors, health and safety, engineering, environmental services, and hospital leadership investigated the cluster as a potential outbreak. Mitigation measures involved a multi-modal response of *C auris* admission screening, weekly point prevalence testing of all BICU patients, environmental surface cultures, enhanced room cleaning, staff education, hand hygiene and personal protective equipment usage audits.

**Results:**

11 cases of *C auris* were identified on our BICU from 7/1/21 - 2/28/22. 5 (45.5%) cases from clinical isolates, 1 (9.1%) from a BICU point prevalence screen on 12/15/21, 5 (45.5%) as a part of weekly point prevalence screens started on 1/1/22. 6 (54.5%) patients were admitted for burn related injuries, 2 (18.2%) for Steven Johnson Syndrome, 2 (18.2%) for necrotizing wounds, and 1 (9.1%) for COVID-19 pneumonia. Cases occurred in 6/10 rooms in the Burn ICU. One (9.1%) patient expired during this outbreak (not deemed to be from *C auris* infection).

**Conclusion:**

Resilient infections like *C auris* pose a risk of nosocomial transmission and potential high morbidity and mortality to burn patients with impaired immune defense and large open wounds. A multidisciplinary team using targeted interventions including screening, education, enhanced cleaning eradicated the outbreak in our BICU. As of 5/1/22, we went 13 + weeks without identifying a new case. The last patient with *C auris* was discharged 3/16/22. To prevent future outbreaks, we created a standardized response plan and instituted a universal screening protocol for *C auris* targeting all patient admissions from skilled nursing facilities and admissions to all ICUs.

**Disclosures:**

**Jorge Paiva Parada, MD MPH**, Shionogi: Honoraria.

